# Decolorization and biodegradation of remazol reactive dyes by *Clostridium* species

**DOI:** 10.1007/s13205-015-0335-0

**Published:** 2016-01-09

**Authors:** Sanmuga Priya Ekambaram, Senthamil Selvan Perumal, Umayal Annamalai

**Affiliations:** Department of Pharmaceutical Technology, Anna University BIT Campus, Tiruchirappalli, 620 024 Tamil Nadu India

**Keywords:** Tirupur, Dyeing effluent, Decolorization, Remazol reactive dyes, Biodegradation

## Abstract

Decolorisation and biodegradation efficacy of potential strains isolated from dyeing effluent collected from Tirupur region, Tamil Nadu, India were studied in remazol reactive dyes. Two potential strains *Clostridium butyricum* (EI05) and *Clostridium acetobutylicum* (EI25) identified by biochemical tests in our previous study were studied for their decolorising efficiency on various remazol reactive dyes (Remazol Blue RGB, Remazol Blue RR, Remazol Navy RGB and Remazol Orange RR). The synthetic dyes showed complete decolorization after 24–72 h by two potential strains EI05 and EI25. *Clostridium acetobutylicum* (EI25) was found to be the most potential strain isolated. The spectral analysis was performed by UV–Visible spectroscopy and FT-IR spectroscopy to study biodegradation. The peak disappearance in UV spectrum, peak shifts and disappearance in FTIR spectrum of treated samples indicated biodegradation. Thus *Clostridium* species could able to decolorize the remazol reactive dyes.

## Introduction

Textile manufacturing units uses a wide range of chemicals in the dyeing process. During the dyeing process it has been reported that 10 % of the dye stuffs used remain unfixed from the fibers and are therefore present unaltered in the effluent, leading to serious environmental pollution (Pearce et al. 2003). The dyeing units are water consuming, and generate a large quantity of wastewater as effluent. Textile industry is one of the fast evolving industries in India, and their rapid growth leads to serious environmental problems especially from bleaching and dyeing units like agriculture issues, rise of heavy metals in ground water, drastic effects on flora and fauna in the surrounding area, etc. Out of various methods of textile effluent treatment for decolourisation including physicochemical methods like adsorption, filtration, coagulation and chemical flocculation, some of the methods were found to be quite effective but expensive. The volume of wastes generated during the industrial treatment process also contributes towards the selection of decolorisation methods. Bioremediation is one of the cheapest techniques, environmentally friendly alternative to other methods and produces less sludge for colour removal (Olukanni et al. 2005).

Azo dyes are of great concern because dye precursors or their biotransformation products such as aromatic amines show carcinogenic and mutagenic effects (Alves de Lima et al. 2007). Azo dyes are the largest class of dye used in the textile industry but due to their structural properties, they are not easily degradable under natural conditions and thus, are not typically removed from water by conventional wastewater treatment systems (Wamik et al. 1998). Considerable research has been done and various microorganisms has been reported to bioremediate remazol reactive dyes (Shah et al. 2013; Joe et al. 2011; Rajendran et al. 2011). Although several reports provide evidence that bacteria degrade azo compounds but very little is known about the biodegradation characteristics of these compounds. Thus the present study is designed to identify a potential strain with higher efficiency to perform biodegradation. Based on the higher usage rate of the remazol reactive dyes in the Tirupur industries for dyeing purpose, the study was designed to isolate and characterize potential biodegrading indigenous bacteria from the effluent itself and to evaluate its efficacy in terms of decolourisation of the remazol reactive dyes used in the textile industries.

## Materials and methods

Dyes were obtained from Dystar India Pvt. Ltd., Mumbai. UV–Visible spectrophotometer—Shimadzu Pharmaspec UV-1700, FT-IR—Spectrum RX I, Perkin Elmer was used for spectral characterization.

Isolation and identification of potential strains from the effluent was reported in our previous study (Sanmuga priya et al. 2014). The selected potential strains as per our previous study was taken to decolorize remazol reactive dyes.

Each dye solution was screened for *λ*_max_ in the UV–Visible spectrophotometer and noted. Ten percent freshly prepared inocula of selected bacterial isolates in broth (at the mid log phase) was added to the 25 mL of the prepared dye (25 mg/mL concentration) in the test tube. 5 mL of the sample was withdrawn aseptically for assaying every 24 h. The withdrawn sample was centrifuged at 7830 rpm for 10 min and the supernatant liquid is used for assay in UV–Visible spectrophotometer. The optical density (OD) values were noted at its *λ*_max_. The decolourization studies were conducted at 38 °C for 96 h and the optical density values were taken every 24 h (Prasad et al. 2011).

The pure dye sample at 25 ppm concentration was analysed through UV–Visible spectrophotometer and the spectrum was recorded. Simultaneously the decolourised samples were also analysed through Shimadzu UV–Visible spectrophotometer and the spectrum was overlaid with the control (Ali et al. 2009).

Biodegradation was characterised by FT-IR Spectroscopy. The functional group characterization of the dyes before and after decolourisation was studied. The pure dye solid sample at 25 ppm concentration was mixed with potassium bromide and the pressed pellets were used for analysis. The liquid samples were directly analysed by placing a drop on the thin film cell. IR spectrum was recorded. The samples prepared at the same concentration were inoculated with the desired microbial sample to perform decolourisation and the sample was centrifuged at 13,000 rpm for 30 min. The supernatant was analysed in the mid infrared region 400–4000 cm^−1^ through FT-IR spectroscopy and the spectrum was recorded (Phugare et al. 2011). FT-IR spectrum was interpreted.

## Results and discussion

From our previous study the potential strains decolourising the dye effluent were found to be *Clostridium butyricum* (EI05) and *Clostridium acetobutylicum* (EI25) (Sanmuga priya et al. 2014). Thus these two strains were taken for the decolorisation study of the remazol dyes. The *λ*_max_ of the synthetic dyes were found to be 519 nm for RB-RGB and RB-RR whereas 410 nm for RN-RGB and 419 nm for RO-RR. The percentage decolorisation of the synthetic dyes by the selected two microorganisms EI05 and EI25 were given in Tables 1 and 2. 100 % decolorisation was achieved after 48 h for RB-RGB and RB-RR by all the organisms. Within 24 h time period the organism EI25 have shown the maximum efficiency of 87.2 and 95.1 % decolorisation for RB-RGB and RB-RR respectively. From Table 2 it is clear that 100 % decolorisation was achieved for RN-RGB at 48 h and RO-RR at 72 h by both the organisms. The maximum decolorisation (89.4 % for RN-RGB and 84.4 % for RB-RR) was shown at 24 h for RN-RGB and RB-RR. The decolorisation rate of Remazol RR decreased at 48 h by EI05 and EI25 followed by complete decolorisation at 72 h. This marked reduction in decolorisation at 48 h and followed by increase in decolorisation rate at 72 h needs further investigation on the decolorisation mechanism. To the best of our knowledge, this is the first report on *Clostridium* species on bioremediation of remazol dyes.

**Table 1:** Decolorization studies of Remazol Blue RGB and Remazol Blue RR dyes

Organism	% Decolorization in							
	24 h		48 h		72 h		96 h	
	RB-RGB	RB-RR	RB-RGB	RB-RR	RB-RGB	RB-RR	RB-RGB	RB-RR
EI05	93.1	84.1	97.7	96.0	100	100	100	100
EI25	96.7	78.5	100	97.6	100	100	100	100

**Table 2:** Decolorization studies of Remazol Navy RGB and Remazol Orange RR dyes

Organism	% Decolorization in							
	24 h		48 h		72 h		96 h	
	RN-RGB	RO-RR	RN-RGB	RO-RR	RN-RGB	RO-RR	RN-RGB	RO-RR
EI05	82.9	91.3	94.5	100	100	100	100	100
EI25	60.6	92.6	84.9	100	100	100	100	100

Remazol blue was completely decolorised up to 100 % by *Rhizopus arrhizus* (Ulkuye et al. 2013) after 48 h. Remazol navy blue was biosorbed with *Pseudomonas putida* in an aqueous solution (Ratnamala and Brajesh 2013) and decolorised by *Proteus* sp. SUK7 (Patil et al. 2012). Biotransformation of remazol orange 3R was studied using *Pseudomonas aeruginosa* and the dye was decolorized up to 98 % within 15 min (Surwase et al. 2013). In the present study *Clostridium* strains were identified as a potent bacterium for bioremediation of remazol dyes. Compared to the previous results, the effect of the microbe EI25 on RN-RGB, RO-RR, RB-RGB and RB-RR was found to be satisfactory, anyhow the detailed mechanism involved in the biodegradation and the effect of various factors on rate of decolorisation has to be studied in future.

**Fig. 1:**
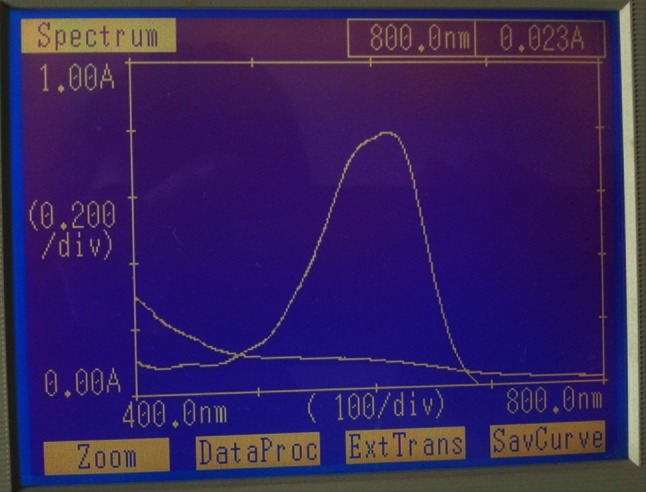
UV spectrum of treated and untreated RB-RGB

**Fig. 2:**
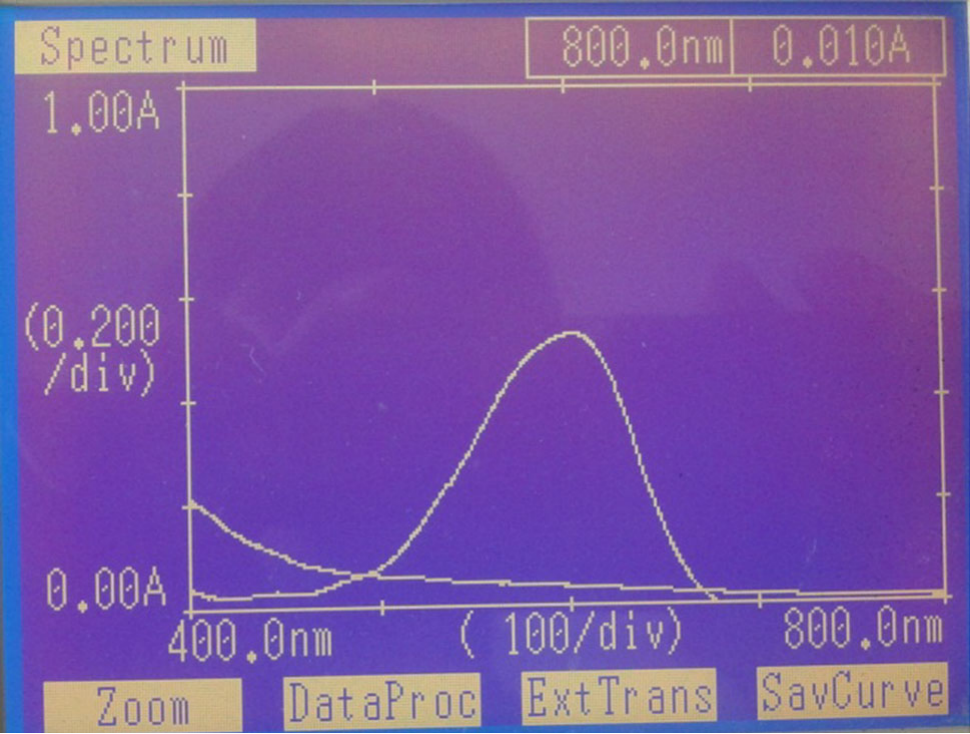
UV spectrum of treated and untreated RB-RR dye

**Fig. 3:**
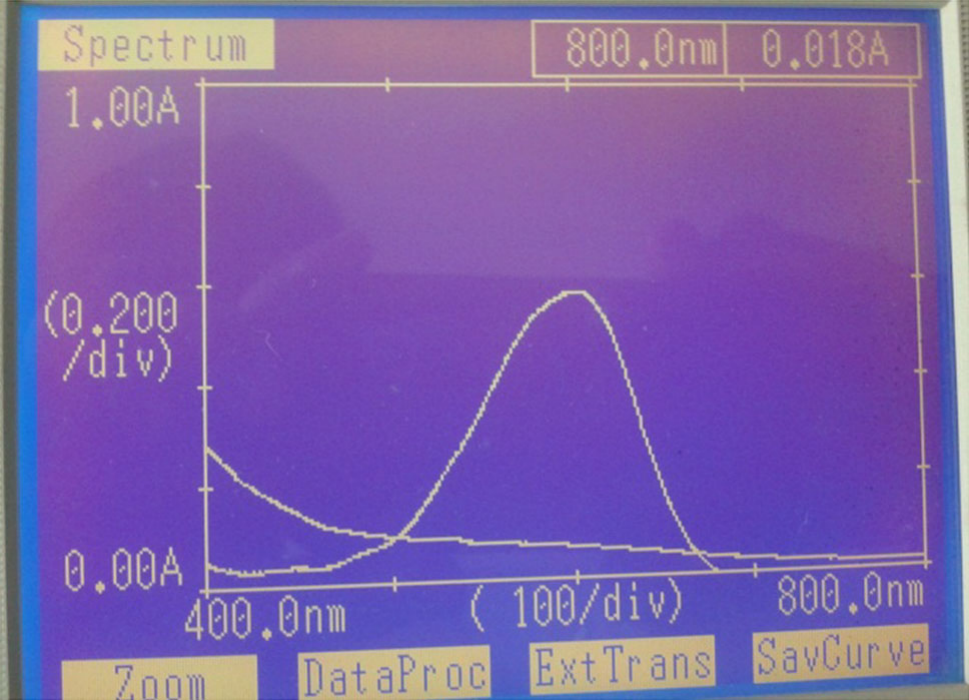
UV spectrum of treated and untreated RN-RGB dye

**Fig. 4:**
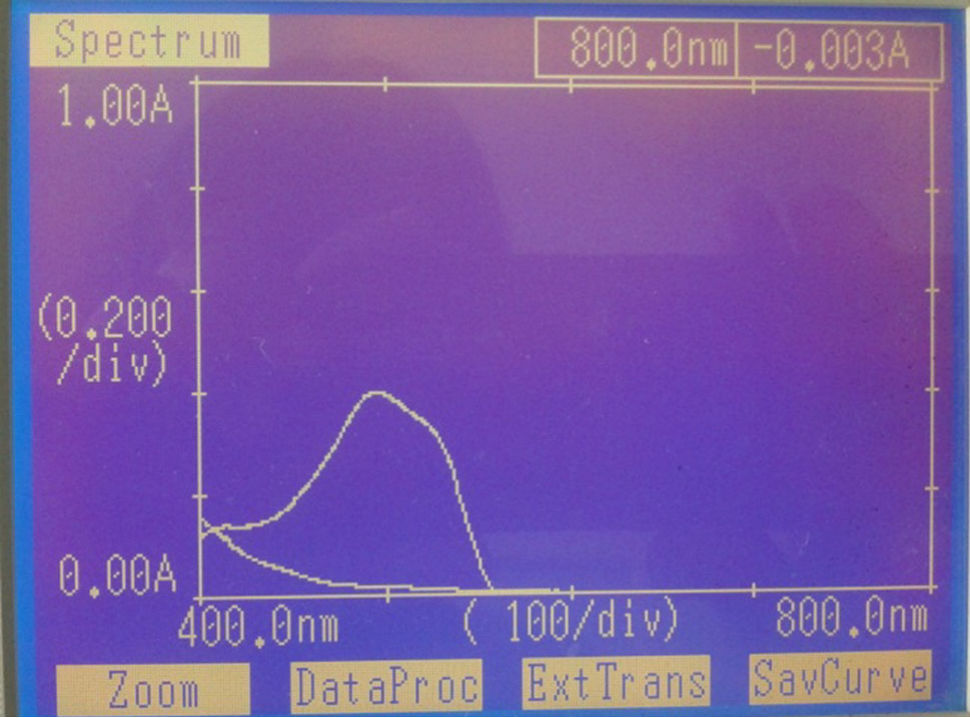
UV spectrum of treated and untreated RO-RR dye

The spectral analysis was performed using UV–Visible spectrophotometer and FT-IR spectroscopy. EI05 and EI25 were studied for decolourisation of the dyes and spectral analysis has been performed. The spectrum of control dye and the spectrum of the decolorised sample were overlaid and recorded. From spectral analysis by UV spectrophotometer, the peak present in the control dye was reduced drastically and the peak disappears at its *λ*_max_ after decolorisation in all the dyes (Figs. 1, 2, 3, 4). Thus stands the proof of decolourisation.

**Fig. 5:**
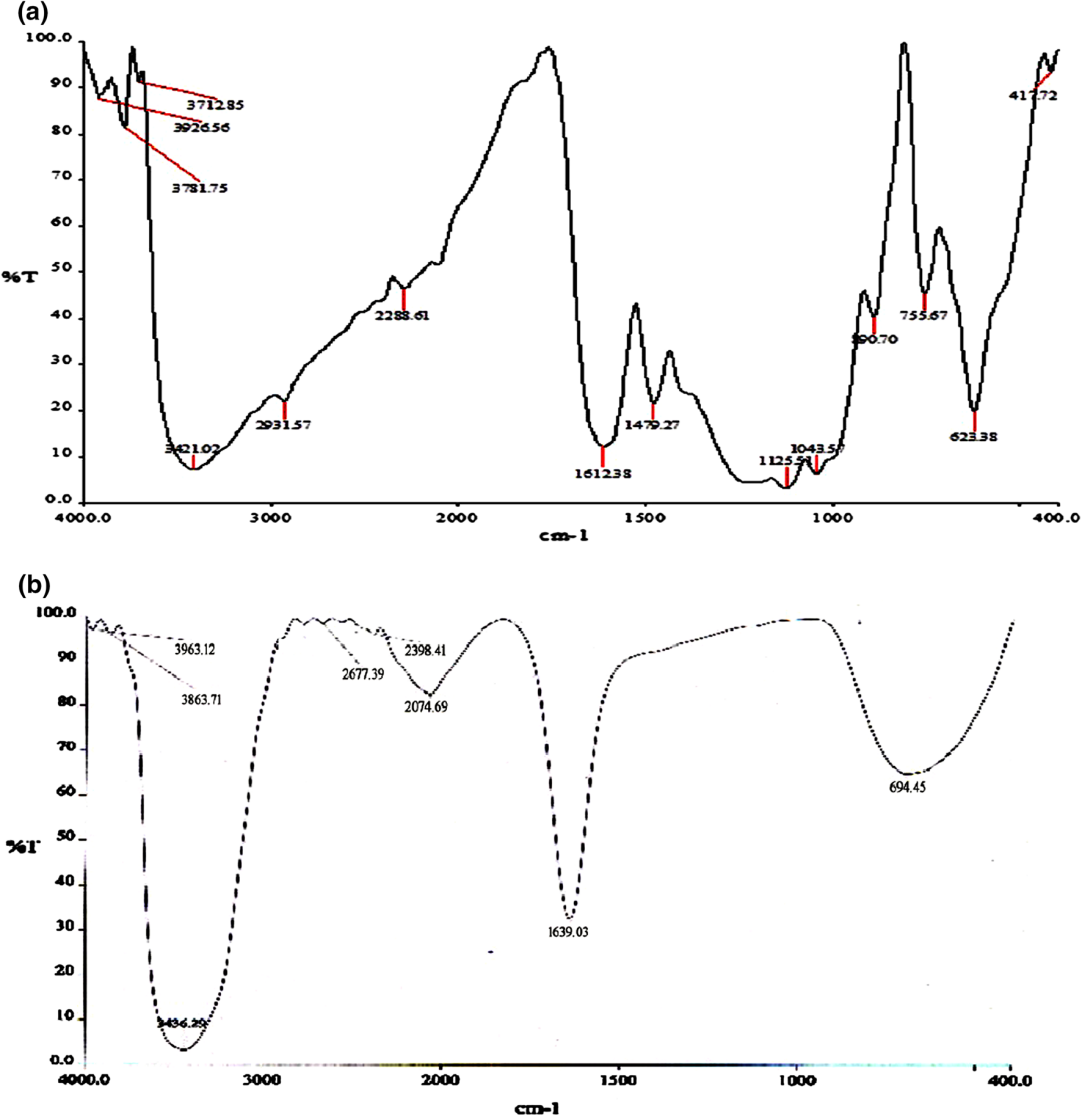
FT-IR spectrum of control dye (**a**) and treated (**b**) RB-RGB dye

**Fig. 6:**
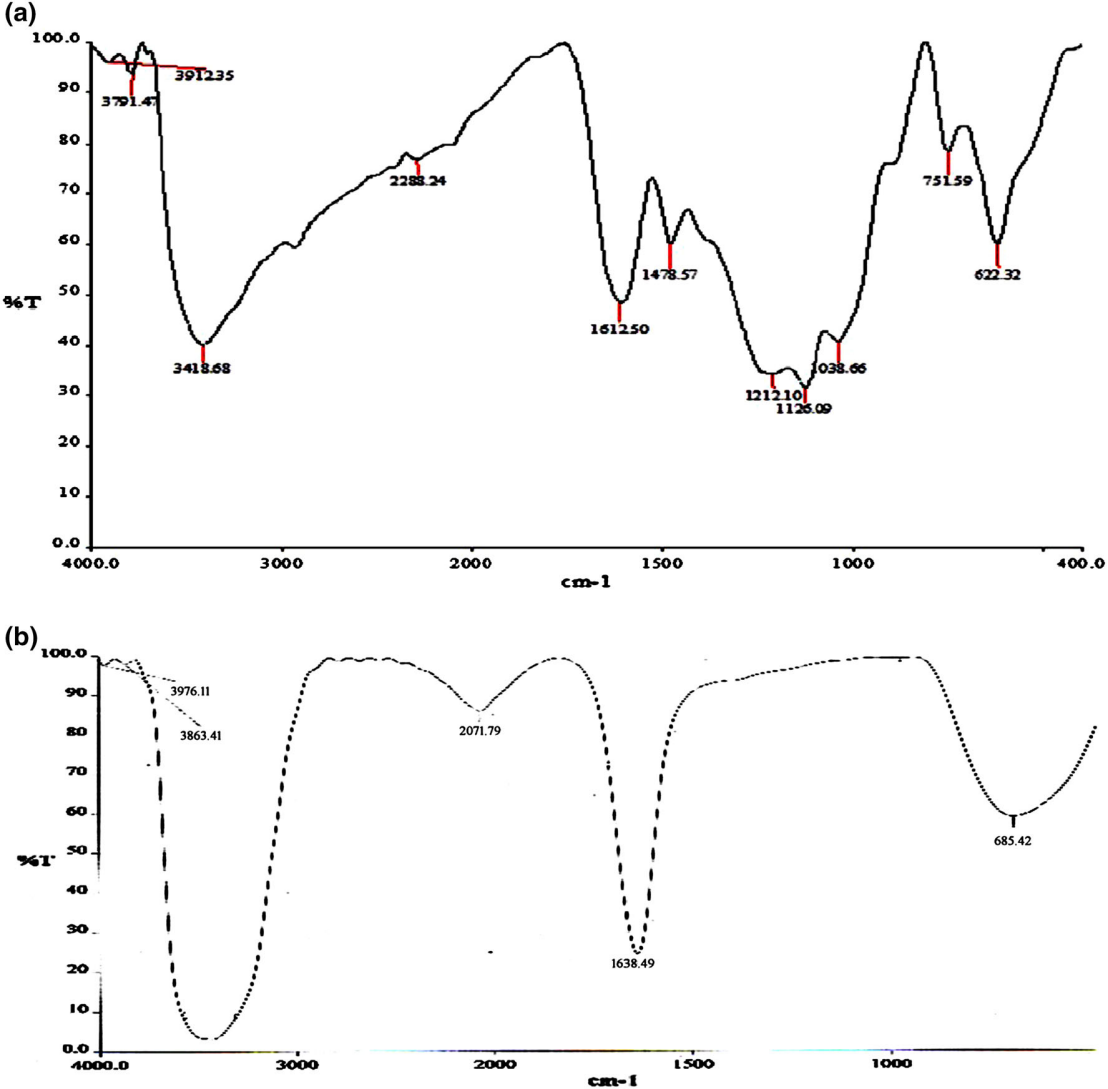
FT-IR spectrums of control dye (**a**) and treated (**b**) RB-RR dye

**Fig. 7:**
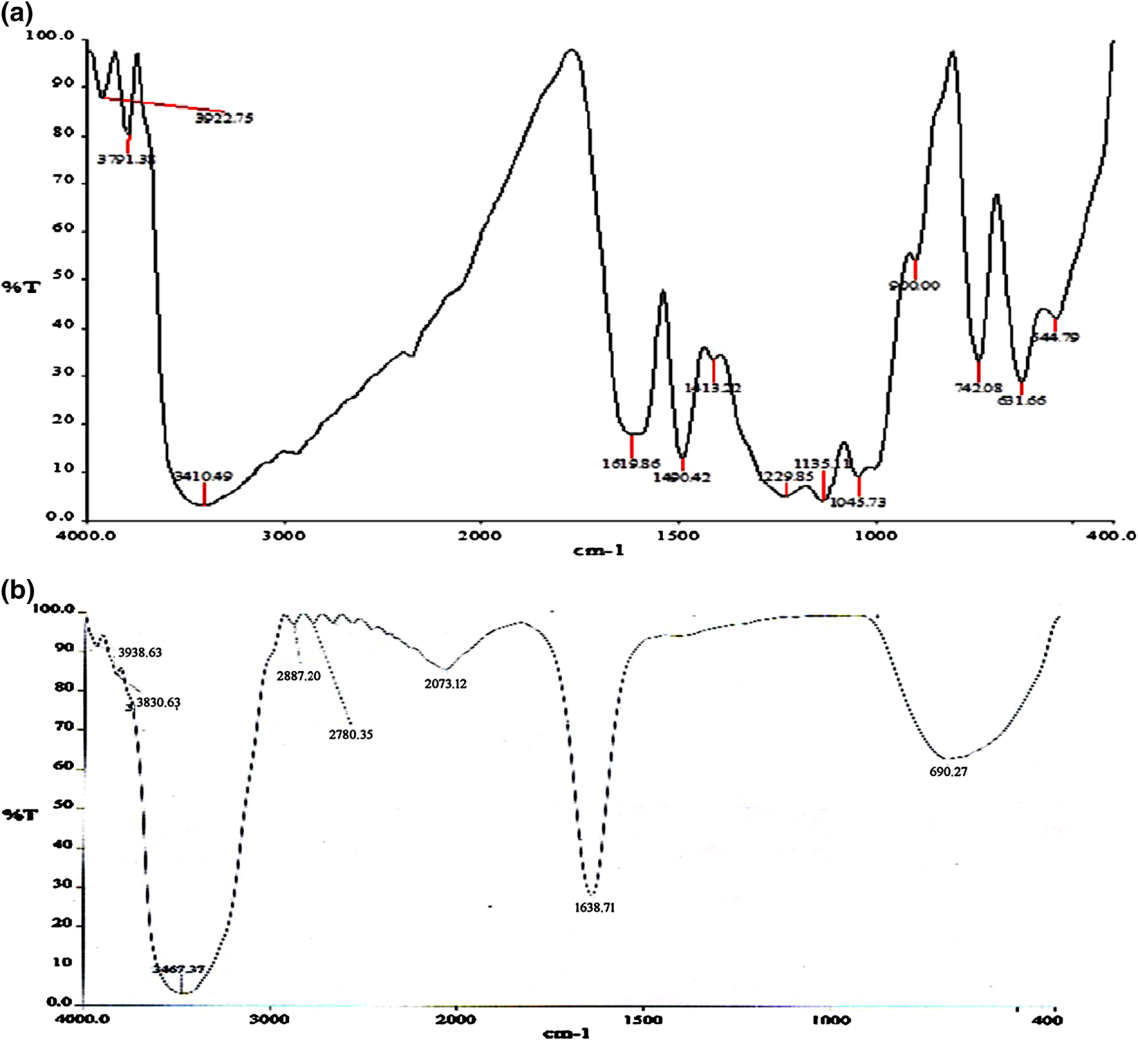
FT-IR spectrum of control (**a**) and treated (**b**) RN-RGB dye

**Fig. 8:**
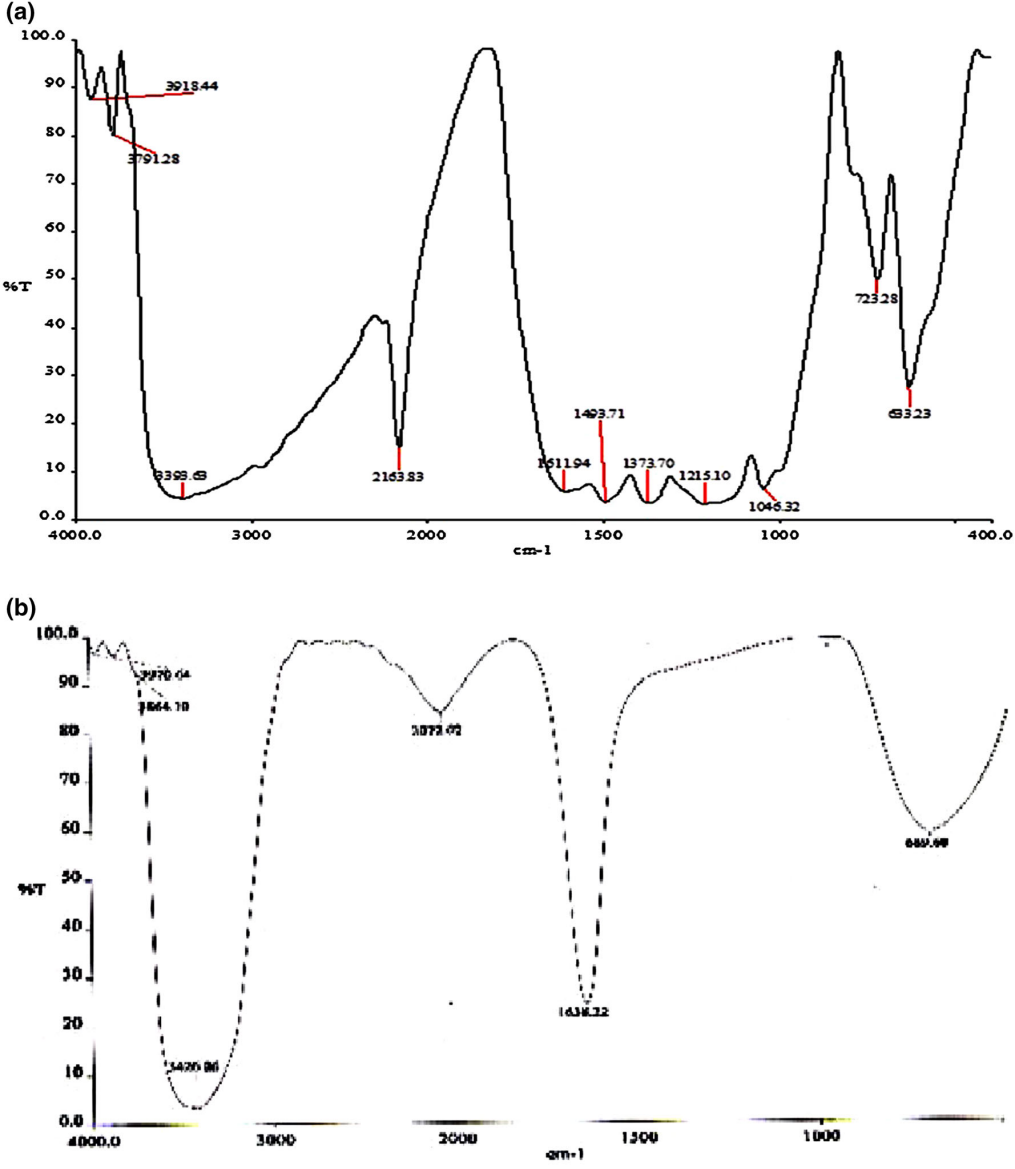
FT-IR spectrum of control (**a**) and treated (**b**) RO-RR dye

The spectrum of the control dye and decolorised samples was recorded with FT-IR spectroscopy. Figures 5, 6, 7 and 8 show the spectrum recorded from the control and treated dyes of RB RGB, RB RR, RN RGB and RO RR respectively. Compared to the control dye spectrum, the FT-IR spectrum of the decolorised sample showed a significant change in the position of peaks. From Fig. 5a the peaks in the control dye spectrum of RB-RGB represented the stretching vibrations of N–H bond (secondary amides) at 3421 cm^−1^, C–H stretching of alkanes at 2931.57 cm^−1^, N=N stretching of azo compounds at 1612.38 cm^−1^, N–H trans stretching of secondary amides at 1479.27 cm^−1^, S=O asymmetric stretching of sulfones at 1125.54 cm^−1^, C–H out of plane bending at 696.43 cm^−1^. The FTIR spectrum of treated RB-RGB (Fig. 5b) showed major peaks at 3436.29 cm^−1^ for N–H stretching, 2074.69 cm^−1^ for C–H stretching of alkanes, 1639.03 cm^−1^ for acetamide group, 694.45 cm^−1^ for C–H out of plane bending. All other peaks disappeared in the dye metabolites. The FT-IR spectrum of RB-RR dye (Fig. 6a) showed major peaks at 3418.68 cm^−1^ (N–H stretching), 1612.50 cm^−1^ (N=N stretching of azo compounds), 1478.57 cm^−1^ (N–H trans stretching of secondary amides), 1125 (S=O asymmetric stretching of sulfones), 622.32 cm^−1^ (C–H bending vibrations). The treated dye metabolites (Fig. 6b) showed shift in peaks (3422.16, 1638.49 and 685.42 cm^−1^) as well as disappearance of peaks.

The control dye RN-RGB (Fig. 7a) showed a broad peak at 3410.49 cm^−1^ indicating N–H stretching vibration, sharp peaks at 1619.86 cm^−1^ (N=N stretching of azo compounds) and 1490.42 cm^−1^ (N–H trans stretching of secondary amides), 1135.17 cm^−1^ (S=O asymmetric stretching of sulfones), 742.08, 631.66 cm^−1^ for C–H bending vibrations. The treated dye (Fig. 7b) showed shift in peak at 3467.37, 1638.71 and 690.27 cm^−1^. All other sharp peaks got disappeared in the treated dye sample. RO-RGB peak pattern in untreated control dye (Fig. 8a) showed peaks at 3393.63 cm^−1^ (N–H stretching vibration), 2163.83 and 1611.94 cm^−1^ (N=N stretching of azo compounds), 1493.71 cm^−1^(N–H trans stretching of secondary amides), 1373.70 cm^−1^ (CH_3_ bending absorption), 1215.10 cm^−1^ (S=O asymmetric stretching of sulfones), 723.28 and 633.23 cm^−1^. The treated dye (Fig. 8b) showed only three major peaks at 3426.86, 2072.02, 1638.22 and 669.60 cm^−1^.

The change in peak pattern as well as disappearance of few peaks in the treated samples after degradation when compared to the control dye spectrum showed that biodegradation of the dye has occurred.

## Conclusion

The present study was conducted in an effort to identify best indigenous decolorizing microbe for the treatment of dye effluent and reactive dyes in Tirupur region, Tamil Nadu, India. EI05 and EI25, the potential dye decolourising bacteria identified in our previous study was studied on four different types of remazol dyes decolourisation. All the four dyes were found to be decolourised within 48 h of EI25 treatment. The biodegradation of the dyes was characterized by UV–Visible and FTIR spectroscopy. In UV–Visible spectrum the clear disappearance of the peak at its respective *λ*_max_ and significant variation in the peak pattern shown by FTIR spectrum of the Remazol dyes by EI25 (*Clostridium acetobutylicum*) indicates the biodegradation of the dyes. The present study concludes that bioremediation of textile dyes by *Clostridium* species can be developed as an effective, economical and eco friendly method of dye effluent treatment. Further investigations on the factors influencing the biodegradation, kinetics and mechanism involved have to be studied.
